# Nouveau Traite Theorique et Pratique de l'Art du Dentiste

**Published:** 1842-12

**Authors:** 


*Nouveau Traité Theorique et Pratique de l'Art du Dentiste.* Par J. Lefoulon, 8vo. pp. 545. Paris, 1841.

The above is the title of a treatise on the teeth, issued from the French press. The first and second parts of the work are devoted to dental anatomy, physiology, hygiene, pathology and therapeutics, and are merely a recapitulation of the doctrines of the old surgeons and dentists of the French school. The part which treats upon the anatomy and physiology of the teeth, is principally taken from the works of Delabarre, Blandin and Cloquet.

The author however, in his "*new treatise,*" mentions one of the new mysteries of our art, which is, his "*valuable aluminous ethereal paste.*" Like many of the remedies of the present day, it cures every disease; it is very useful in ulcerated and other kinds of diseased gums, and in odontalgia it is "a most valuable remedy." In speaking of this last affection, M. Lefoulon says, "after repeated trials of all the various means, I have finally found this remedy simple and certain;" but unfortunately for suffering humanity, the author does not acquaint us with the ingredients of his paste.

The chapter devoted to the method of filing teeth, contains some useful hints, and is worthy of attention.

The third part, treats on practical dentistry, but the directions there laid down would serve, in the present improved state of the art, but poorly as a guide to the student.

